# Fermented *Aronia melanocarpa* pomace improves the nutritive value of eggs, enhances ovarian function, and reshapes microbiota abundance in aged laying hens

**DOI:** 10.3389/fmicb.2024.1422172

**Published:** 2024-06-19

**Authors:** Zhihua Li, Binghua Qin, Ting Chen, Xiangfeng Kong, Qian Zhu, Md. Abul Kalam Azad, Yadong Cui, Wei Lan, Qinghua He

**Affiliations:** ^1^Department of Food Science and Engineering, College of Chemistry and Environmental Engineering, Shenzhen University, Shenzhen, China; ^2^Hunan Provincial Key Laboratory of Animal Nutritional Physiology and Metabolic Process, National Engineering Laboratory for Pollution Control and Waste Utilization in Livestock and Poultry Production, Institute of Subtropical Agriculture, Chinese Academy of Sciences, Changsha, China; ^3^School of Biology and Food Engineering, Fuyang Normal University, Fuyang, China

**Keywords:** aged laying hens, nutritive value, fermented *Aronia melanocarpa* pomace, ovarian function, microbiota

## Abstract

**Introduction:**

There is a decline in the quality and nutritive value of eggs in aged laying hens. Fruit pomaces with high nutritional and functional values have gained interest in poultry production to improve the performance.

**Methods:**

The performance, egg nutritive value, lipid metabolism, ovarian health, and cecal microbiota abundance were evaluated in aged laying hens (320 laying hens, 345-day-old) fed on a basal diet (control), and a basal diet inclusion of 0.25%, 0.5%, or 1.0% fermented *Aronia melanocarpa* pomace (FAMP) for eight weeks.

**Results:**

The results show that 0.5% FAMP reduced the saturated fatty acids (such as C16:0) and improved the healthy lipid indices in egg yolks by decreasing the atherogenicity index, thrombogenic index, and hypocholesterolemia/hypercholesterolemia ratio and increasing health promotion index and desirable fatty acids (*P* < 0.05). Additionally, FAMP supplementation (0.25%−1.0%) increased (*P* < 0.05) the ovarian follicle-stimulating hormone, luteinizing hormone, and estrogen 2 levels, while 1.0% FAMP upregulated the *HSD3B1* expression. The expression of *VTG II* and *ApoVLDL II* in the 0.25% and 0.5% FAMP groups, *APOB* in the 0.5% FAMP group, and *ESR2* in the 1% FAMP group were upregulated (*P* < 0.05) in the liver. The ovarian total antioxidant capacity was increased (*P* < 0.05) by supplementation with 0.25%−1.0% FAMP. Dietary 0.5% and 1.0% FAMP downregulated (*P* < 0.05) the *Keap1* expression, while 1.0% FAMP upregulated (*P* < 0.05) the *Nrf2* expression in the ovary. Furthermore, 1.0% FAMP increased cecal acetate, butyrate, and valerate concentrations and Firmicutes while decreasing Proteobacteria (*P* < 0.05).

**Conclusion:**

Overall, FAMP improved the nutritive value of eggs in aged laying hens by improving the liver–blood–ovary function and cecal microbial and metabolite composition, which might help to enhance economic benefits.

## 1 Introduction

Eggs are excellent sources of nutrients, such as proteins, lipids, and micronutrients, which are highly beneficial to the physical health of humans (Kovacs-Nolan et al., 2005). However, laying hens are afflicted by a series of physiological health complications, resulting in a decline in their egg-laying performance and egg quality with age. Lipid precursor substances in egg yolk are mainly derived from the liver. The yolk formation and deposition of laying hens are largely determined by the multifunction of the liver–blood–ovary (LBO) axis, including lipid metabolism and hormone regulations of various forms (Wu et al., 2023). The recession activities of the liver and ovary in aged laying hens arise from endocrine disorders and decrease in antioxidant capacity and follicle formation (Gu et al., 2021; Wu et al., 2023). Ovarian dysfunction in laying hens results in the decline of egg-laying performance and quality of eggs, for which decrease in antioxidant capacity is one of the main reasons (Bao et al., 2022). Moreover, modulation of gut microbiota is a prospective approach to alleviating the common problems of aged laying hens, such as the decline in the quality and nutritive value of eggs (Feng et al., 2021; Dai et al., 2022). Hence, strengthening the LBO and gut functions in aged laying hens can contribute to improving their egg-laying performance and quality.

Recently, numerous natural plants or their extracts have been employed as feed additives to promote the production of eggs in aged laying hens. *Aronia melanocarpa*, known as black chokeberry, originated from North America and spread to European and other regions, especially in Poland, with a yearly yield of 14,000–15,000 tons of fresh fruits (Jurikova et al., 2017) and 600–700 tons in China (Ren et al., 2022). It is mainly industrially processed as different food products, such as juice, jams, and dietary supplements for humans (Sidor and Gramza-Michalowska, 2019). After production and processing, it produces large amounts of pomace. *Aronia melanocarpa* pomace (AMP) contains phenolic phytochemicals, dietary fiber, free glucose, fructose, and other bioactive substances (Schmid et al., 2020). It has been reported that AMP can serve as a feed additive to increase production in animals (Lipinska et al., 2017; Ren et al., 2022). AMP contains a relatively high content of fiber. Meanwhile, avians lack enzymes that degrade non-starch polysaccharides, which may affect the adequate absorption of AMP (Erinle and Adewole, 2022). Microbial fermentation helps to enhance the nutritional quality of agricultural by-products as alternative feed ingredients (Cui et al., 2023) and increases the content and bioavailability of dietary polyphenols (Du and Myracle, 2018; Esatbeyoglu et al., 2023). Fermentation of AMP may improve its nutritive value by decreasing fruit wastes generated in the food industry and feed costs in animal husbandry, which is environmentally friendly and economical. However, studies regarding the influence of the fermented AMP (FAMP) on the production performance, quality and nutritive value of eggs in aged laying hens, and the potential mechanisms of FAMP's action remain unclear. Hence, this study explored the effects of the addition of FAMP to aged laying hens' diet on the production performance, quality and nutritive value of eggs, ovarian function, and cecal microbiota and metabolite composition, which might lay the theoretical foundation for improving the nutritive value of eggs due to the FAMP additive.

## 2 Materials and methods

### 2.1 Fermentation of *Aronia melanocarpa* pomace

Dried and cleaned AMP was obtained from Fuyang Fruit Wine Engineering Technology Center (Fuyang, China). FAMP was prepared according to the following procedure. A compound starter culture was prepared to ferment the raw material (AMP). The compound starter culture was composed of a microbial agent mixture, enzyme, medium 1, medium 2, and sucrose at a mass ratio of 1:1:1:1:1. Among them, the microbial agent mixture (Guangdong Microbial Culture Collection Center, Guangzhou, China) contained *Bacillus subtilis* GDMCC 1.372, *Bacillus licheniensis* GDMCC 1.182, *Lactobacillus plantarum* GDMCC 1.648, and *Rhodotorula benthica* GDMCC 2.215 at a mass ratio of 1:1:1:1. The active enzyme was a mixture of cellulase and papain at a mass ratio of 1:1. Medium 1 consisted of cultivated ginseng leaves, which were dried and crushed to a 40-mesh powder. Medium 2 was soybean flour. The above compound starter culture and AMP were blended at a mass ratio of 1:100. The fermentation conditions were as follows: fermentation temperature, 75°C; dissolved oxygen, 3–10%; moisture content, 30–80%; and fermentation method, solid fermentation. The measured nutrient levels (%; dry matter basis) of FAMP were as follows: ash, 5.20; crude protein, 10.63; ether extract, 4.80; crude fiber, 15.90; calcium, 0.55; total phosphorus, 0.28; and gross energy, 18.65 MJ/kg. Amino acid and fatty acid profiles of FAMP are presented in Supplementary Tables S1 and S2, respectively.

### 2.2 Animals, diets, and treatments

A total of 320 Yukou Jingfen No. 8 laying hens that were 345 days old with similar health conditions were selected and randomly allocated into one of the four treatment groups with eight replicates per treatment (ten birds/replicate). The four treatment groups were fed on a basal diet with the addition of 0 (control, CON), 0.25%, 0.5%, and 1.0% FAMP, respectively. The basal diet for laying hens was formulated based on the standard nutriment requirements (Supplementary Table S3). The trial continued for eight weeks and there was a 1-week pre-feeding trial. All laying hens were raised in a controlled environment at 18–24 °C, humidity of 45–60%, and 16 h/day of illumination. The other feeding management followed routine commercial feeding management protocols during the trial.

### 2.3 Determination of laying performance

The weight and number of eggs and feed intake in each replicate were recorded during the whole trial. Then, average laying rate (ALR), average egg weight (AEW), average daily feed intake (ADFI), and feed-to-egg ratio (FER) were analyzed from weeks 1 to 2, 3 to 4, 5 to 6, 7 to 8, and 1 to 8 of the trial.

### 2.4 Egg quality analysis

Three eggs per replicate were randomly collected for egg quality measurement every two weeks during the trial. The egg shape index was determined by a Vernier caliper and calculated using previous formulas described by Feng et al. (2021). The eggshell strength, egg weight, albumen height, Haugh unit, and yolk color (Roche colorimetric unit) were measured with an Egg Force Reader and an EggAnalyzer (ORKA Food Technology Ltd., Ramat HaSharon, Israel), respectively. Then, the eggshell, yolk, and albumen were separated, weighed, and their percentages calculated. The eggshell thickness was measured by a hand-held micrometer (Deli Group, Ningbo, China).

### 2.5 Sample collection and plasma preparation

Three eggs from each replicate were collected to obtain the egg yolk and egg albumen for further nutritive value analyses after the 8-week animal trial. The plasma samples were obtained through wing vein-blood collection and centrifugation and kept at −80°C for plasma hormone, biochemical parameter, and antioxidant indicator analyses. Then, hens from each replicate were euthanized by jugular vein bleeding. The liver and ovary were dissected and kept at −80°C for mRNA extraction, and the ovary samples were also collected for hormone and antioxidant indicator analyses. The cecal contents were collected to determine the microbiota abundance and short-chain fatty acids (SCFA).

### 2.6 Count of ovarian follicles

In hens' ovaries, numbers of large white (2–5 mm), small yellow (6–8 mm), and hierarchical (>9 mm) follicles were recorded based on previously described methods (Li et al., 2020; Huang et al., 2021).

### 2.7 Determination of ether extract concentration and fatty acid profiles in yolks

The ether extract level of egg yolks was determined by a Soxhlet extraction with petroleum. The fatty acid composition was determined by employing the area normalization method (Hu et al., 2017). Briefly, total lipids were taken from egg yolks following the chloroform–methanol process. The fatty acid methyl ester was obtained with KOH/methanol and determined by a gas chromatograph (GC2030, Shimadzu Corporation, Kyoto, Japan).

According to the percentage of particular fatty acids of egg yolks, saturated fatty acids (SFA), polyunsaturated fatty acids (PUFA), monounsaturated fatty acids (MUFA), n-3 PUFA, and n-6 PUFA percentages were counted as follows: SFA = C14:0 + C16:0 + C17:0 + C18:0, MUFA = C16:1n-7 + C18:1n-9 + C20:1n-9, n-3 PUFA = C18:3n-3 + C22:6n-3, n-6 PUFA = C18:2n-6 + C18:3n-6 +C20:2n-6 + C20:3n-6 + C20:4n-6, and PUFA= n-3 PUFA + n-6 PUFA. Meanwhile, the health lipid indices related to fatty acids, such as atherogenicity index (AI), thrombogenic index (TI), hypocholesterolemic/hypercholesterolaemic ratio (H/H), health promotion index (HPI), and desirable fatty acids (DFA), were calculated as reported in our previous study (Li et al., 2024): AI = (C12:0 + 4 × C14:0 + C16:0)/UFA, TI = (C14:0 + C16:0 + C18:0)/(0.5 × MUFA + 0.5 × n-6 PUFA + 3 × n-3 PUFA + n-3/ n-6 PUFA), H/H ratio = (C18:1n-9 + C18:2n-6 + C20:4n-6 + C18:3n-3 + C20:5n-3 + C22:5n-3+C22:6n-3)/(C14:0 + C16:0), HPI = (MUFA + PUFA)/(C12:0 + 4 × C14:0 +C16:0), and DFA = C18:0 + MUFA + PUFA.

### 2.8 Measurement of crude protein and amino acid content in egg albumen

The crude protein content in egg albumen was evaluated by the Kjeldahl method. The content of amino acids in egg albumen was determined using a previously described method (Hu et al., 2017). Briefly, freeze-dried egg albumen samples were prepared using 6 mol/L hydrochloric acid at 110°C for 24 h. Then, the suspensions were filtered and detected by an ion-exchange AA analyzer (LA8080, Hitachi, Tokyo, Japan).

### 2.9 Analysis of plasma and ovary homogenate hormones

Ovary samples were placed in normal saline, vortexed, and then centrifuged at 4°C for supernatant collection. The protein content in ovary homogenate was measured with a bicinchoninic acid assay kit (Nanjing Jiancheng Bioengineering Institute, Nanjing, China).

The content of estrogen 2 (E_2_), follicle-stimulating hormone (FSH), and luteinizing hormone (LH) in plasma and ovary homogenate was determined using the enzyme-linked immunosorbent assay (ELISA) kits (Hunan Richamp Biotechnology Co., Ltd., Changsha, China). The optical density (OD) values were obtained on a spectrophotometer (Tecan, Infinite M200 Pro, Basel, Switzerland).

### 2.10 Determination of plasma and ovary redox status and plasma biochemical parameters

The content of superoxide dismutase (SOD), glutathione peroxidase (GPX), total antioxidant capacity (T-AOC), glutathione (GSH), and malondialdehyde (MDA) in plasma and ovary homogenate was determined by colorimetry (Nanjing Jiancheng Bioengineering Institute, Nanjing, China) on a spectrophotometer (Tecan, Infinite M200 Pro, Basel, Switzerland). The protein content of each ovary homogenate sample was used to normalize the indexes mentioned above. The content of total cholesterol (TC) and triglyceride (TG), alanine aminotransferase (ALT), and aspartate aminotransferase (AST) in plasma was also determined by colorimetry (Nanjing Jiancheng Bioengineering Institute, Nanjing, China) on a spectrophotometer (Tecan, Infinite M200 Pro, Basel, Switzerland).

### 2.11 RNA extraction and mRNA quantification

The total RNA in both liver and ovary samples was measured using the TransZol agent (TransGen Biotech, Beijing, China). The concentration and purity of extracted RNA samples were evaluated by a Nanophotometer N60 (Implen, GmbH, Germany). The total RNA was reverse transcribed into cDNA following instructions provided in the RT Kit (Accurate Biology, Changsha, China). Quantitative PCR amplification was carried out on the LightCycler R 480II Real-Time PCR System (Roche, Basel, Switzerland) using the qPCR Kit (Accurate Biology, Changsha, China) following the manufacturer's instructions. Characteristic primer sequences devised through the Primer 3 web (https://primer3.ut.ee/) are available in Supplementary Table S4. The relative gene expression was computed with the 2^−ΔΔ*Ct*^ method (Livak and Schmittgen, 2001).

### 2.12 Cecal microbial analysis

The cecal microbiota composition analysis was performed by the Shanghai Personal Biotechnology Co., Ltd., Shanghai, China (Zhu et al., 2022). Briefly, the total microbial DNA of cecal contents was extracted and amplified with specific primers (forward primer: 5′-ACTCCTACGGGAGGCAGCA-3′ and reverse primer: 5′-GGACTACHVGGGTWTCTAAT-3′) to obtain bacterial V3-V4 sequences. After purification and amplification, the PCR products were paired-end sequenced on an Illumina NovaSeq platform (Illumina, San Diego, CA, USA). After quality control and chimera removal, amplified sequence variants (ASVs) were taxonomically aligned with species annotation using the Greengenes database. The α and β diversity indices were analyzed to detect the diversity, richness, and dissimilarity of the microbiota. Kruskal–Wallis test was performed to determine the differential phyla and genera. The linear discriminant analysis (LDA) effect size (LEfSe; LDA ≥ 2, *P* < 0.05) and random forest analysis were applied to identify and distinguish the microbiota in the four groups. The Kyoto Encyclopedia of Genes and Genomes (KEGG) pathway analysis of the microbiota was conducted with the Phylogenetic Investigation of Communities by Reconstruction of Unobserved States (PICRUSt2) method. The correlation between cecal microbiota abundance (top 50 genera) and phenotypes was performed using Spearman correlation by the R package (|R| > 0.50, *P* < 0.05).

### 2.13 Measurement of cecal short-chain fatty acid profile

The concentrations of cecal SCFA were measured according to our previous study (Li et al., 2021) using Agilent 7890A gas chromatograph (Agilent Inc., Palo Alto, CA, USA).

### 2.14 Statistical analysis

All data analyses were performed using the SPSS 22.0 software (SPSS Inc., Chicago, IL, USA) package. The normality and homogeneity of variances of the data were assessed using the Shapiro–Wilk test and Levene's test, respectively. When applicable, the one-way analysis of variance (ANOVA) following Tukey's *post-hoc* test was performed to assess significant differences. Otherwise, Welch's ANOVA and Games-Howell tests were performed for the significant difference analyses. The results are expressed as means with standard error of the mean (SEM). *P* < 0.05 was considered statistically significant, whereas 0.05 ≤ *P* < 0.1 suggested a trend.

## 3 Results

### 3.1 Laying performance and egg quality

Table 1 showed no notable differences (*P* > 0.05) in the ALR, AEW, and FER among the four groups during 1 to 2, 3 to 4, 5 to 6, 7 to 8, and 1 to 8 weeks of the trial. The ADFI was elevated (*P* < 0.05) in the 0.25% and 0.5% FAMP groups relative to that in the CON group during 7–8 weeks. The ADFI in the 0.25% FAMP group was higher (*P* < 0.05) than that of the 1% FAMP group during 7 to 8 weeks, and showed an increasing trend (*P* = 0.082) during 1 to 8 weeks. As presented in Table 2, the addition of 0.25% FAMP decreased (*P* < 0.05) the egg shape index relative to that in the CON and 0.5% AMP groups at week 2. The eggshell thickness was higher (*P* < 0.05) in the 0.25% FAMP group than in the 1% FAMP group at week 6. No notable differences (*P* > 0.05) were found in the eggshell strength, Haugh unit, albumen height, yolk color, and percentages of eggshell, yolk, and albumen among the four groups throughout the trial.

**Table 1:** Effects of FAMP on productive performance of aged laying hens.

**Items**	**CON**	**Dietary FAMP level**			**SEM**	***P*-value**
		**0.25%**	**0.5%**	**1.0%**		
**1 to 2 weeks**						
ALR (%)	71.79	65.63	69.46	70.98	1.555	0.529
AEW (g)	51.10	51.60	51.75	51.19	0.231	0.726
ADFI (g)	95.31	96.09	95.34	95.47	0.454	0.742
FER	2.60	2.89	2.73	2.66	0.064	0.452
**3 to 4 weeks**						
ALR (%)	69.91	70.45	71.43	69.20	1.195	0.934
AEW (g)	52.67	51.31	50.84	51.43	0.287	0.131
ADFI (g)	91.74	90.48	89.33	89.51	0.305	0.023
FER	2.51	2.52	2.50	2.53	0.042	0.997
**5 to 6 weeks**						
ALR (%)	73.21	72.86	70.27	72.50	1.213	0.840
AEW (g)	51.54	51.45	51.47	51.45	0.224	0.999
ADFI (g)	100.75	101.76	101.74	101.68	0.121	0.056
FER	2.69	2.74	2.84	2.75	0.044	0.716
**7 to 8 weeks**						
ALR (%)	71.43	76.03	70.18	75.80	1.306	0.275
AEW (g)	51.01	52.06	52.00	51.76	0.244	0.413
ADFI (g)	97.37^c^	99.46^a^	98.90^ab^	97.93^bc^	0.230	0.003
FER	2.71	2.53	2.72	2.52	0.046	0.243
**1 to 8 weeks**						
ALR (%)	71.58	71.20	70.33	72.12	1.035	0.947
AEW (g)	51.57	51.61	51.50	51.47	0.198	0.995
ADFI (g)	96.29	96.94	96.33	96.15	0.138	0.082
FER	2.62	2.65	2.69	2.60	0.036	0.858

Data are represented as means with SEM; *n* = 8. Values with different lowercase letters within the same row differ significantly (*P* < 0.05). FAMP, fermented *Aronia melanocarpa* pomace; ALR, average laying rate; ADFI, average daily feed intake; AEW, average egg weight; FER, feed-to-egg ratio.

**Table 2:** Effects of FAMP on egg quality of aged laying hens.

**Items**	**CON**	**Dietary FAMP level**			**SEM**	***P*-value**
		**0.25%**	**0.5%**	**1.0%**		
**Week 2**						
Eggshell strength (N)	38.72	42.25	40.35	40.59	0.794	0.499
Shape index	1.33^a^	1.29^b^	1.35^a^	1.33^ab^	0.007	0.007
Albumen height (mm)	3.38	3.70	4.44	3.77	0.157	0.104
Yolk color	13.67	14.04	13.88	13.79	0.122	0.763
Haugh unit	55.04	57.72	64.04	56.82	1.600	0.215
Eggshell thickness (mm)	0.34	0.33	0.34	0.34	0.003	0.548
Eggshell percent (%)	13.34	12.88	12.66	12.91	0.105	0.133
Yolk percent (%)	32.72	32.60	31.91	31.59	0.331	0.588
Albumen percent (%)	53.88	54.56	55.43	55.59	0.377	0.357
**Week 4**						
Eggshell strength (N)	40.74	40.32	40.24	39.84	0.746	0.982
Shape index	1.32	1.34	1.33	1.33	0.005	0.627
Albumen height (mm)	5.17	4.05	4.37	4.31	0.173	0.113
Yolk color	13.96	13.50	13.21	13.31	0.132	0.191
Haugh unit	68.70	61.70	64.59	63.25	1.632	0.488
Eggshell thickness (mm)	0.34	0.33	0.33	0.32	0.002	0.120
Eggshell percent (%)	13.24	13.07	13.06	12.98	0.113	0.890
Yolk percent (%)	30.48	30.97	30.78	30.82	0.304	0.955
Albumen percent (%)	56.29	55.96	56.16	56.21	0.318	0.987
**Week 6**						
Eggshell strength (N)	39.81	43.09	40.65	40.20	0.738	0.408
Shape index	1.36	1.34	1.34	1.33	0.006	0.568
Albumen height (mm)	4.21	5.51	4.94	5.51	0.251	0.217
Yolk color	13.75	14.38	14.17	14.38	0.104	0.104
Haugh unit	63.86	71.31	69.12	71.31	1.731	0.393
Eggshell thickness (mm)	0.344^ab^	0.354^a^	0.345^ab^	0.334^b^	0.002	0.012
Eggshell percent (%)	13.12	13.41	12.96	13.38	0.104	0.368
Yolk percent (%)	31.34	30.94	30.48	30.33	0.237	0.436
Albumen percent (%)	55.54	55.64	56.56	56.30	0.292	0.561
**Week 8**						
Eggshell strength (N)	40.14	39.18	39.80	41.24	0.912	0.891
Shape index	1.32	1.34	1.34	1.34	0.005	0.665
Albumen height (mm)	5.18	5.01	4.89	4.81	0.100	0.618
Yolk color	14.04	14.17	14.10	14.08	0.091	0.973
Haugh unit	71.25	70.11	68.97	68.60	0.959	0.776
Eggshell thickness (mm)	0.33	0.33	0.34	0.34	0.003	0.683
Eggshell percent (%)	13.05	13.24	12.69	13.05	0.101	0.294
Yolk percent (%)	31.25	30.62	31.26	30.37	0.250	0.506
Albumen percent (%)	55.70	56.15	56.04	56.58	0.268	0.732

Data are represented as means with SEM; *n* = 8. Values with different lowercase letters within the same row differ significantly (*P* < 0.05). FAMP, fermented *Aronia melanocarpa* pomace.

### 3.2 The ether extract and fatty acid profiles in the egg yolk

As seen in Table 3, the C16:0 percentage was lower (*P* < 0.05) in the 0.5% FAMP group than that of the CON group. The C20:4n-6 percentage showed a decrease (*P* < 0.05) in the 0.25% FAMP group compared to the CON group. Moreover, FAMP supplementation exhibited a declining trend (*P* = 0.059) in the C20:3n-6 percentage compared to the CON group. Additionally, the SFA percentage and AI and TI indexes were reduced (*P* < 0.05), whereas H/H, HPI, and DFA indexes were elevated (*P* < 0.05) in the 0.5% FAMP group by comparison with the CON group. PUFA, PUFA/SFA, n-3 PUFA, and n-6 PUFA in the four groups showed no obvious distinctions (*P* > 0.05).

**Table 3:** Effects of FAMP on ether extract concentration, fatty acid profile, and health lipid indices of egg yolk.

**Items**	**CON**	**Dietary FAMP level**			**SEM**	***P*-value**
		**0.25%**	**0.5%**	**1.0%**		
Ether extract (g/100g tissue)	33.89	34.55	34.36	34.94	0.309	0.712
C14:0	0.40	0.41	0.38	0.39	0.006	0.419
C16:0	25.78^a^	25.23^ab^	24.55^b^	24.93^ab^	0.146	0.013
C16:1n-7c	3.12	2.88	2.69	2.74	0.076	0.192
C17:0	0.14	0.15	0.17	0.15	0.005	0.282
C18:0	8.26	8.17	8.05	8.11	0.112	0.940
C18:1n-9c	42.90	42.95	44.47	42.60	0.435	0.446
C18:2n-6c	16.26	17.22	16.60	17.90	0.448	0.610
C18:3n-6	0.12	0.13	0.11	0.12	0.004	0.636
C18:3n-3	0.65	0.68	0.70	0.74	0.022	0.594
C20:1n-9c	0.22	0.21	0.21	0.21	0.003	0.538
C20:2n-6	0.13	0.14	0.13	0.14	0.004	0.797
C20:3n-6	0.14	0.12	0.12	0.12	0.003	0.059
C20:4n-6	1.29^a^	1.18^b^	1.22^ab^	1.26^ab^	0.016	0.037
C22:6n-3	0.60	0.54	0.60	0.58	0.010	0.157
**Health lipid indices**						
SFA(%)	34.58^a^	33.96^ab^	33.15^b^	33.59^ab^	0.168	0.010
MUFA(%)	46.23	46.04	47.37	45.55	0.441	0.542
PUFA(%)	19.19	20.00	19.48	20.86	0.482	0.662
PUFA/SFA	0.56	0.59	0.59	0.62	0.016	0.552
n-3 PUFA(%)	1.25	1.22	1.30	1.32	0.028	0.575
n-6 PUFA(%)	17.94	18.78	18.18	19.54	0.461	0.646
n-6/n-3 PUFA	14.32	15.50	13.89	14.80	0.255	0.135
AI	0.42^a^	0.41^ab^	0.39^b^	0.40^ab^	0.003	0.006
TI	0.96^a^	0.94^ab^	0.90^b^	0.91^ab^	0.008	0.022
H/H	2.36^b^	2.44^ab^	2.55^a^	2.50^ab^	0.022	0.007
HPI	2.39^b^	2.46^ab^	2.57^a^	2.51^ab^	0.021	0.008
DFA(%)	73.68^b^	74.21^ab^	74.91^a^	74.52^ab^	0.147	0.015

Data are represented as means with SEM; *n* = 6. Values with different lowercase letters within the same row differ significantly (*P* < 0.05). FAMP, fermented *Aronia melanocarpa* pomace; SFA, saturated fatty acids; PUFA, polyunsaturated fatty acids; MUFA, monounsaturated fatty acids; AI, atherogenicity index; TI, thrombogenic index; H/H, hypocholesterolaemic-to-hypercholesterolaemic ratio; HPI, health promotion index; DFA, desirable fatty acids.

### 3.3 Crude protein and amino acid content in egg albumen

The valine content in egg albumen was reduced (*P* < 0.05) in the 0.25% FAMP group than in the CON group. The 0.25% and 0.5% FAMP groups exhibited a decreasing trend with regard to the content of histidine (*P* = 0.093), proline (*P* = 0.060), and branched-chain amino acid (BCAA, *P* = 0.089) of egg albumen relative to the CON group (Table 4).

**Table 4:** Effects of FAMP on crude protein and amino acid content of egg albumen.

**Items, %**	**CON**	**Dietary FAMP level**			**SEM**	***P*-value**
		**0.25%**	**0.5%**	**1.0%**		
Crude protein	9.93	9.27	9.60	9.50	0.154	0.535
EAA	5.11	4.60	4.75	4.92	0.091	0.227
Arginine	0.57	0.51	0.52	0.55	0.010	0.249
Histidine	0.23	0.21	0.21	0.22	0.004	0.093
Isoleucine	0.53	0.47	0.48	0.50	0.010	0.118
Leucine	0.86	0.77	0.80	0.83	0.015	0.160
Lysine	0.65	0.60	0.60	0.66	0.012	0.173
Methionine	0.49	0.46	0.52	0.46	0.015	0.496
Phenylalanine	0.62	0.56	0.58	0.61	0.011	0.162
Threonine	0.46	0.42	0.43	0.45	0.008	0.187
Valine	0.69^a^	0.60^b^	0.61^ab^	0.65^ab^	0.012	0.025
NEAA	4.83	4.34	4.43	4.64	0.082	0.153
Alanine	0.61	0.55	0.56	0.59	0.011	0.131
Aspartic acid	1.06	0.95	0.96	1.01	0.018	0.104
Glutamic acid	1.37	1.23	1.25	1.31	0.023	0.129
Glycine	0.36	0.32	0.33	0.35	0.006	0.139
Proline	0.34	0.30	0.30	0.33	0.006	0.060
Serine	0.70	0.64	0.65	0.68	0.012	0.272
Tyrosine	0.39	0.35	0.38	0.38	0.007	0.387
TAA	9.94	8.94	9.18	9.56	0.173	0.189
FAA	3.97	3.56	3.62	3.79	0.068	0.135
BCAA	2.08	1.84	1.89	1.98	0.037	0.089

Data are represented as means with SEM; *n* = 6. Values with different lowercase letters within the same row differ significantly (*P* < 0.05). FAMP, fermented *Aronia melanocarpa* pomace; EAA, essential amino acid, containing arginine, histidine, isoleucine, leucine, lysine, methionine, phenylalanine, threonine, tryptophan, and valine; NEAA, non-essential amino acid, containing alanine, aspartic acid, cystine, glutamic acid, glycine, proline, serine, and tyrosine; TAA, total amino acid; FAA, flavor amino acid, containing aspartic acid, glutamic acid, glycine, alanine, and arginine; BCAA, branched-chain amino acid, containing isoleucine, leucine, and valine.

### 3.4 The number of ovarian follicles, hormone levels of plasma and ovary, and hormone synthesis and receptor genes in the ovary

**Figure 1:**
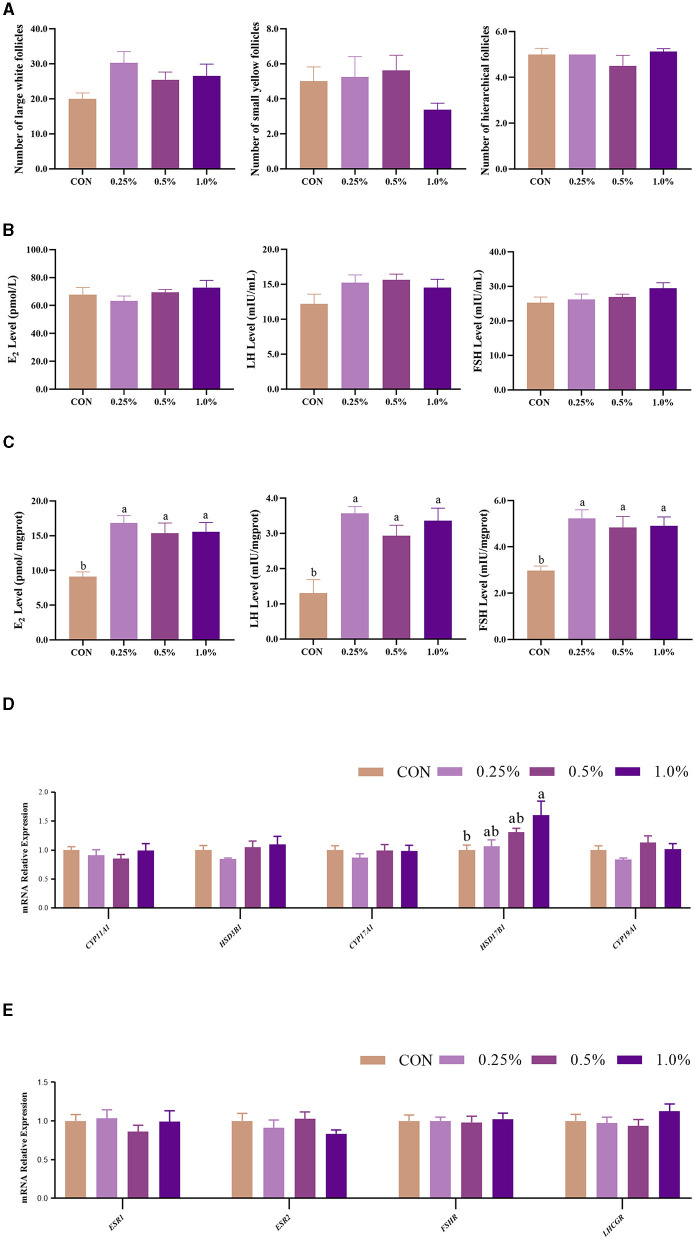
FAMP affected the number of follicles, hormone levels, and gene expression related to hormone synthesis/receptors. The number of follicles **(A)**, hormone levels in plasma **(B)** and ovary **(C)**, and gene expression related to hormone synthesis **(D)** and receptor **(E)** of the ovary. The hens in the CON, 0.25%, 0.5%, and 1.0% groups were fed the basal diet supplemented with 0, 0.25%, 0.5%, and 1.0% fermented *Aronia melanocarpa* pomace (FAMP), respectively. Data are represented as means with SEM; *n* = 8. Values with different lowercase letters in the histogram differ significantly (*P* < 0.05). *CYP11A1*, cytochrome P450 family 11 subfamily A member 1; *HSD3B1*, 3 beta- and steroid delta-isomerase 1; *CYP17A1*, cytochrome P450 family 17 subfamily A member 1; *HSD17B1*, hydroxysteroid 17-beta dehydrogenase 1; *CYP19A1*, cytochrome P450 family 19 subfamily A member 1; *ESR1*, estrogen receptor 1; *ESR2*, estrogen receptor 2; *FSHR*, follicle stimulating hormone receptor; *LHCGR*, luteinizing hormone/choriogonadotropin receptor.

The results of ovarian follicles are shown in Figure 1A. The number of large white follicles in the FAMP groups exhibited an increasing trend (*P* = 0.092) relative to the CON group. There were no significant changes (*P* > 0.05) in plasma FSH, LH, and E_2_ contents among the four groups (Figure 1B). However, ovary FSH, LH, and E_2_ levels were higher (*P* < 0.05) in the 0.25%, 0.5%, and 1.0% FAMP groups relative to the CON group (Figure 1C). The ovary *HSD17B1* expression was upregulated (*P* < 0.05) in the 1.0% FAMP group relative to the CON group. The mRNA expression involved in hormonogenesis (including *CYP11A1, HSD3B1, CYP17A1*, and *CYP19A1*) and hormone receptors (including *ESR1, ESR2, FSHR*, and *LHCGR*) in ovarian tissues did not differ among the four groups (Figures 1D, E).

### 3.5 Plasma biochemical indicators and expression of genes involved in egg yolk precursor synthesis and transport

**Figure 2:**
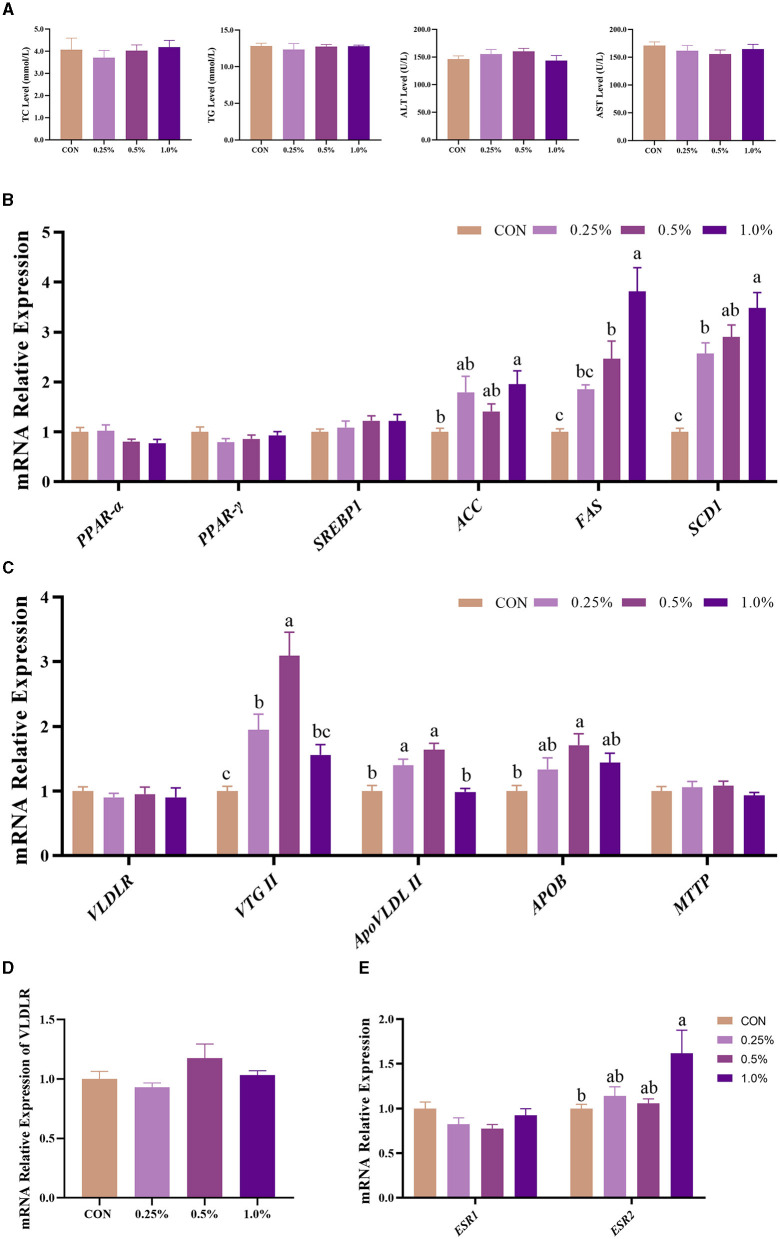
FAMP affected plasma biochemical indicators and egg yolk precursors synthesis and transport of the liver. The plasma biochemical indicators **(A)** and expression of genes related to egg yolk precursor synthesis **(B)** and transport **(C)** of the liver. The mRNA expression of *VLDLR* of the ovary **(D)** and *ESR1* and *ESR2* of the liver **(E)**. The hens in the CON, 0.25%, 0.5%, and 1.0% groups were fed the basal diet supplemented with 0, 0.25%, 0.5%, and 1.0% fermented *Aronia melanocarpa* pomace (FAMP), respectively. Data are represented as means with SEM; *n* = 8. Values with different lowercase letters in the histogram differ significantly (*P* < 0.05). *ACC*, acetyl-CoA carboxylase; *APOB*, apolipoprotein B; *ApoVLDL II*, apo very low-density lipoprotein II; *ESR1*, estrogen receptor 1; *ESR2*, estrogen receptor 2; *FAS*, fatty acid synthase; *MTTP*, microsomal triglyceride transfer protein; *PPAR*α, peroxisome proliferator-activated receptor alpha; *PPAR*-γ, peroxisome proliferator-activated receptor gamma; *SCD1*, stearoyl-CoA desaturase 1; *SREBP1*, sterol regulatory element binding protein 1; TC, total cholesterol; TG, triglyceride; *VLDLR*, very low-density lipoprotein receptor; *VTG* II, vitellogenin 2.

As shown in Figure 2A, plasma TC, TG, ALT, and AST levels of the four groups showed no significant differences (*P* > 0.05). The gene expression involved in the synthesis of yolk precursor (including *ACC, FAS*, and *SCD1*) and *ESR2* in the liver was upregulated (*P* < 0.05) in the 1.0% FAMP group compared with the CON group (Figures 2B, E). The gene expressions of the liver-respecting egg yolk precursor transportation (including V*TG II, ApoVLDL II*, and *APOB*) were upregulated (*P* < 0.05) in the 0.5% FAMP group relative to the CON group (Figure 2C). As for the *VLDR* gene expression of the ovary, no difference (*P* > 0.05) was observed among the four groups (Figure 2D).

### 3.6 Plasma and ovary redox status and the *Keap1/Nrf2* pathway of the ovary

**Figure 3:**
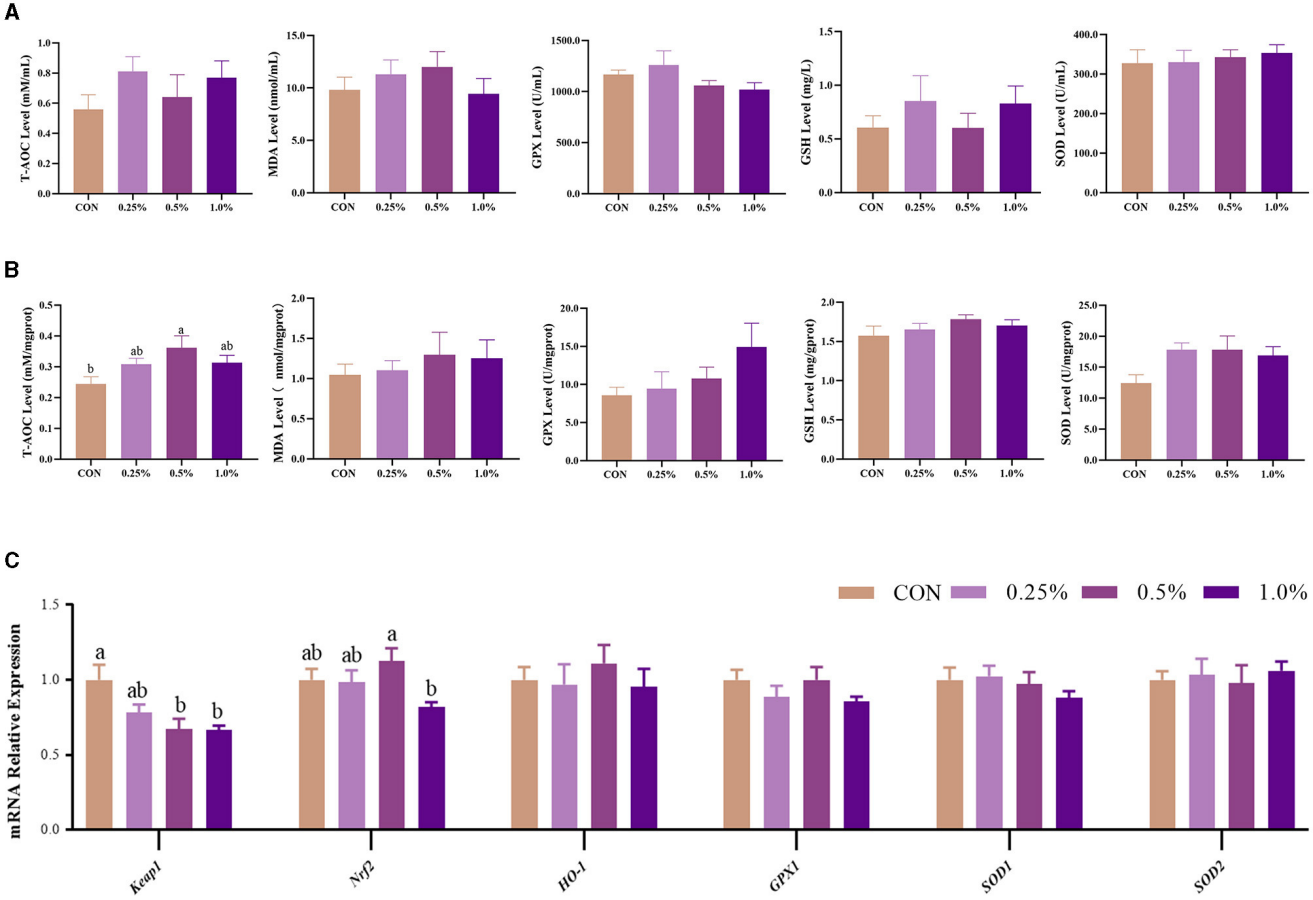
FAMP-affected the antioxidant capacity of the plasma and ovary. The plasma **(A)** and ovary **(B)** redox level and gene expression in the Keap1/Nrf2 signaling pathway of the ovary **(C)**. The hens in CON, 0.25%, 0.5%, and 1.0% groups were fed the basal diet supplemented with 0, 0.25%, 0.5%, and 1.0% fermented *Aronia melanocarpa* pomace (FAMP), respectively. Data are represented as means with SEM; *n* = 8. Values with different lowercase letters in the histogram differ significantly (*P* < 0.05). T-AOC, total antioxidant capacity; SOD, superoxide dismutase; GSH, glutathione; GPX, glutathione peroxidase; MDA, malondialdehyde; *Keap1*, Kelch-like ECH-associated protein 1; *Nrf2*, NF-E2-related factor 2; *HO-1*, heme oxygenase 1; *GPX1*: glutathione peroxidase 1; *SOD1*, superoxide dismutase 1; *SOD2*, superoxide dismutase 2.

The levels of plasma redox status-related parameters, including T-AOC, GSH, GSH-PX, SOD, and MDA, were not changed (*P* > 0.05) by dietary FAMP supplementation (Figure 3A). In the ovary, T-AOC (*P* < 0.05) and SOD (*P* = 0.064) levels were increased in the 0.5% FAMP group in comparison to the CON group (Figure 3B). Furthermore, the *Keapl* expression of the 0.5% and 1.0% FAMP groups was decreased (*P* < 0.05) relative to the CON group. Moreover, the *Nrf2* expression of the 0.5% FAMP group was higher (*P* < 0.05) than in the 1.0% FAMP group.

### 3.7 Microbiota structure and community in cecal contents

**Figure 4:**
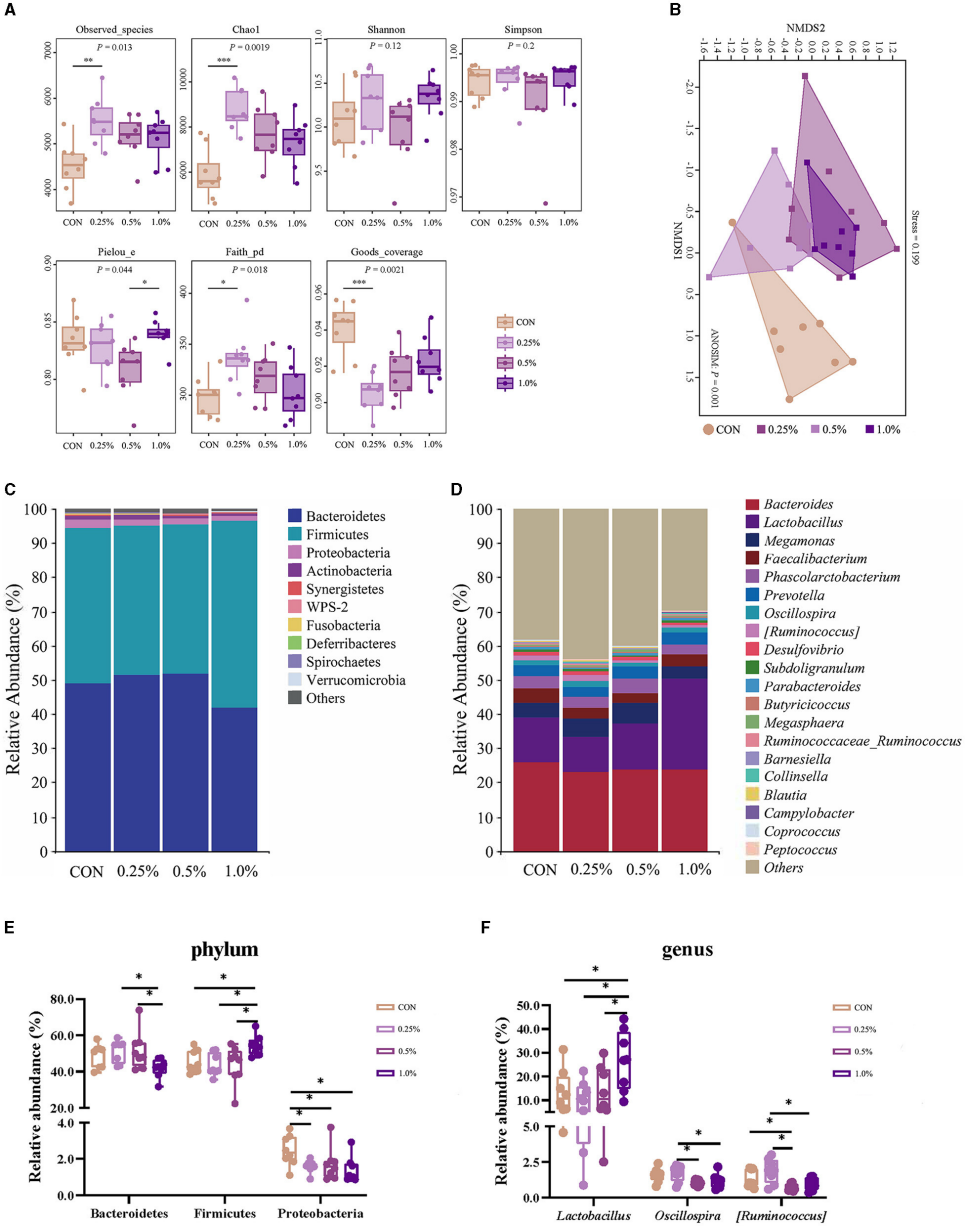
FAMP affected the cecal microbiota composition. The α- **(A)** and β-diversity **(B)** indices and community composition of cecal microbiota of aged laying hens at the phylum **(C, E)** and genus **(D, F)** levels. The hens in the CON, 0.25%, 0.5%, and 1.0% groups were fed the basal diet supplemented with 0, 0.25%, 0.5%, and 1.0% fermented *Aronia melanocarpa* pomace (FAMP), respectively. Data are represented as means with SEM; *n* = 8. *P < 0.05.

The results of the α-diversity are presented in Figure 4A. The Observed_species, Chao1, and Faith-pd indexes were higher, whereas the Goods coverage index was lower in the 0.25% FAMP group relative to the CON group (*P* < 0.05). The Pielou_e index of the 1.0% FAMP group was higher (*P* < 0.05) relative to the 0.5% FAMP group. The β-diversity analysis (non-metric multidimensional scaling ordination plot) indicated that cecal microbial communities were differentiated between the CON and the three FAMP groups (Figure 4B). Bacteroidetes, Firmicutes, and Proteobacteria predominated in the cecal phyla (Figure 4C). An increase (*P* < 0.05) in the relative abundance of Bacteroidetes was found in the 0.25% and 0.5% FAMP groups relative to the 1.0% FAMP group. The relative abundance of Firmicutes was increased (*P* < 0.05) in the 1.0% FAMP group relative to the other three groups. Especially, we found a decrease (*P* < 0.05) in the relative abundance of Proteobacteria in the FAMP groups in comparison to the CON group (Figure 4E). Furthermore, the top ten genera were *Bacteroides, Lactobacillus, Megamonas, Faecalibacterium, Phascolarctobacterium, Prevotella, Oscillospira, [Ruminococcus], Desulfovibrio*, and *Subdoligranulum* (Figure 4D). Notably, the relative abundance of *Lactobacillus* of the 1.0% FAMP group was the highest (*P* < 0.05) among all groups. The 0.5% and 1% FAMP groups exhibited a decrease in the relative abundances of *Oscillospira* and *[Ruminococcus]* (*P* < 0.05) relative to the 0.25% FAMP group. The relative abundance of *[Ruminococcus]* of the 0.5% FAMP group was decreased (*P* < 0.05) relative to the CON group (Figure 4F).

### 3.8 Cecal microbiota biomarkers and function prediction

**Figure 5:**
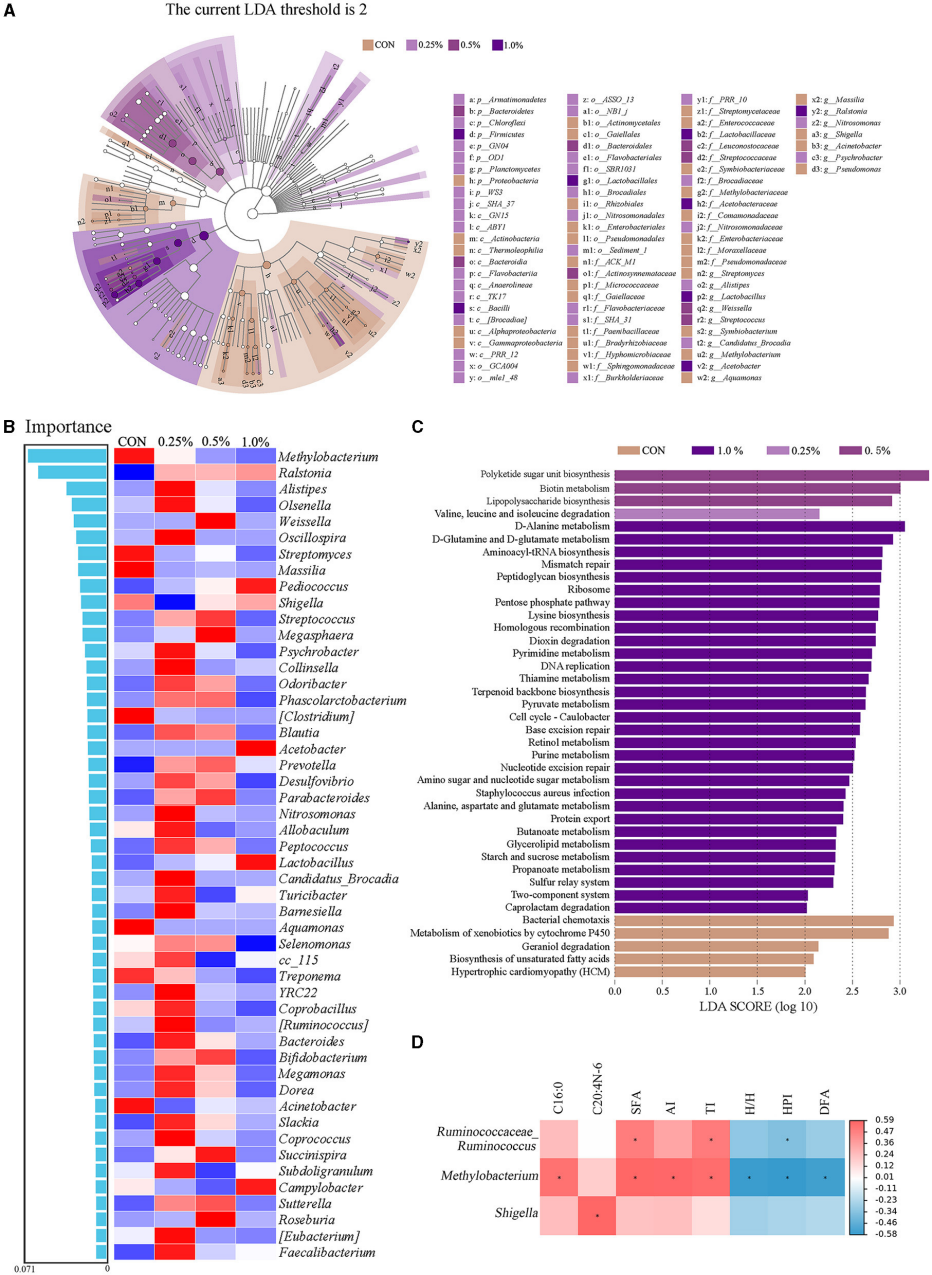
FAMP affected the predicted microbiota functions in cecal contents. Linear discriminant analysis effect size (LEfSe analysis, LDA score ≥ 2) for taxonomic abundance analysis **(A)**, random forest for identifying microbial markers **(B)**, different enrichment pathways for predicted function **(C)**, and correlation analysis (|R| > 0.50, *P* < 0.05) between microbiota (top 50 genera) and differential fatty acid indices **(D)** of cecal microbiota of aged laying hens. The hens in the CON, 0.25%, 0.5%, and 1.0% groups were fed the basal diet supplemented with 0, 0.25%, 0.5%, and 1.0% fermented *Aronia melanocarpa* pomace (FAMP), respectively.

As presented in Figure 5A, the LEfSe analysis of taxa with LDA score ≥ 2 showed that Proteobacteria in the CON group; Armatimonadetes, Chloroflexi, GNO4, OD1, Planctomycetes, and WS3 in the 0.25% FAMP group, Bacteroidetes in the 0.5% FAMP, and Firmicutes in the 1.0% FAMP group were enriched. At the genus level, *Streptomyces, Symbiobacterium, Methylobacterium, Massilia, Shigella, Acinetobacter*, and *Pseudomonas* in the CON group, *Alistipes, Candidatus_Brocadia, Nitrosomonas*, and *Psychrobacter* in the 0.25% FAMP group, *Weissella* and *Streptococcus* in the 0.5% FAMP group, and *Lactobacillus, Acetobacter*, and *Ralstonia* in the 1.0% FAMP group were enriched. The random forest model was performed to further identify the biomarkers. As shown in Figure 5B, the top five marker genera among the four groups were *Methylobacterium, Ralstonia, Alistipes, Olsenella*, and *Weissella* at the genus level. Meanwhile, the 0.25% FAMP group had increased *Alistipes, Olsenella, Oscillospira*, and others. Furthermore, the pathways at level 3 were further identified (Figure 5C). The results showed that the CON, 0.25%, 0.5%, and 1.0% FAMP groups significantly enriched 5, 1, 3, and 31 pathways, respectively. There were five significantly enriched pathways in the CON group, namely, bacterial chemotaxis, metabolism of xenobiotics by cytochrome P450, geraniol degradation, biosynthesis of unsaturated fatty acids, and hypertrophic cardiomyopathy. The pathway enriched in the 0.25% FAMP group was valine, leucine, and isoleucine degradation. There were three significantly enriched pathways in the 0.5% FAMP group, namely polyketide sugar unit biosynthesis, biotin metabolism, and lipopolysaccharide biosynthesis. The 1.0% FAMP group enriched 31 pathways, and the top 10 pathways included D-alanine metabolism, D-glutamine and D-glutamate metabolism, aminoacy1-tRNA biosynthesis, mismatch repair, peptidoglycan biosynthesis, ribosome, pentose phosphate pathway, lysine biosynthesis, homologous recombination, and dioxin degradation.

### 3.9 Correlations between microbiota and differential fatty acid indices of egg yolk

As shown in Figure 5D, the positive correlations were obtained between *Ruminococcaceae_Ruminococcus* with SFA and TI, *Methylobacterium* with C16:0, SFA, AI, and TI, and *Shigella* with C20:4n-6 (|R| > 0.50, *P* < 0.05). The negative correlations (|R| > 0.50, *P* < 0.05) were obtained between *Ruminococcaceae_Ruminococcus* with HPI, and *Methylobacterium* with H/H, HPI, and DFA.

### 3.10 Cecal short-chain fatty acid composition

**Figure 6:**
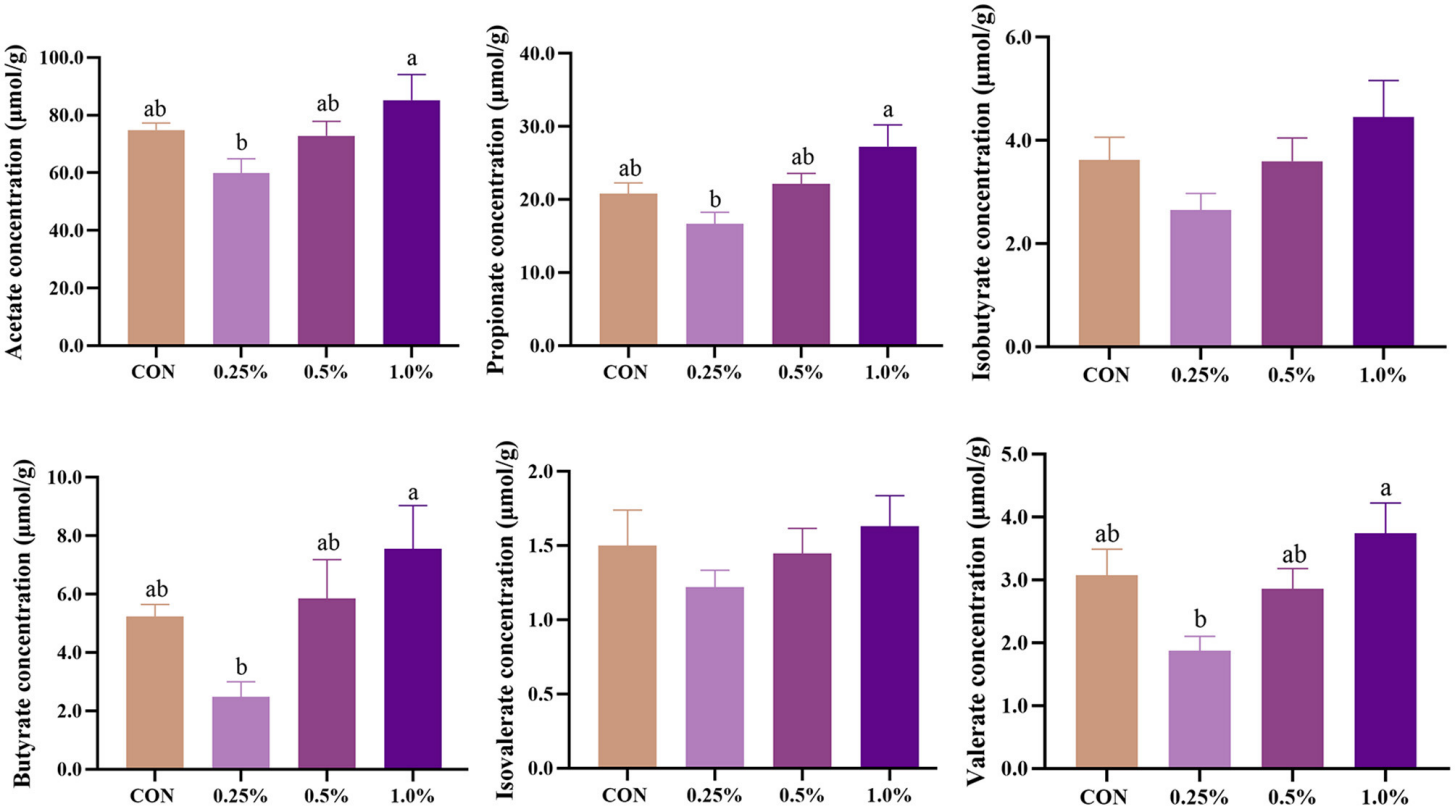
FAMP affected the concentrations of short-chain fatty acids in cecal content. The hens in CON, 0.25%, 0.5%, and 1.0% groups were fed the basal diet supplemented with 0, 0.25%, 0.5%, and 1.0% fermented *Aronia melanocarpa* pomace (FAMP), respectively. Data are represented as means with SEM; *n* = 8. Values with different lowercase letters in the histogram differ significantly (*P* < 0.05).

The levels of cecal SCFA of aged laying hens are presented in Figure 6. The levels of acetate, propionate, butyrate, and valerate were increased (*P* < 0.05) in the 1.0% FAMP group relative to the 0.25% FAMP group. Dietary 0.25%−1.0% FAMP did not (*P* > 0.05) affect the level of isobutyrate.

## 4 Discussion

Increasing evidence suggests that a reduction in performance and egg quality influences the nutritive and economic value of eggs in aged hens after high-intensity metabolism at the peak laying period (He et al., 2023), which refers to the stage after 48 weeks of age, accounting for about half of the entire laying period. Therefore, modulating the aging of the ovary is essential to enhance the egg quality of aged laying hens. In this trial, we observed the addition of FAMP improved the health lipid indices of yolk and nutritive value of eggs, enhanced lipid metabolism in the LBO axis, regulated the reproductive hormone metabolism, increased ovarian antioxidant function and cecal SCFA, and reshaped cecal microbiota composition in Yukou Jingfen No. 8 laying hens at 50 and 58 weeks of age. Thus, the findings support the reuse of fermented AMP, a byproduct of the food industry, in the feed of laying hens to enhance the nutritive value of eggs.

Laying performance and egg quality are important indicators in the poultry industry, and these parameters are dependent on the diverse organs participating in reproduction. Aging can alter the body's genome, proteome, and metabolome, leading to diverse damages and metabolic dysfunctions, hindering the normal function of multiple organs, such as the ovary, liver, and intestine (Li G. et al., 2023). Our results showed that FAMP (0.25%−1.0%) supplementation did not significantly improve the laying performance (including ALR, AEW, and FER) and egg quality (e.g., yolk color, Haugh unit, albumen height, and other indicators). Similarly, supplementation of 3% dried AMP (Sosnówka-Czajka and Skomorucha, 2021), 2.5% AMP (Loetscher et al., 2014), and 4% grape pomace (Kara et al., 2016) in laying hens' diets also had no obvious effects on laying performance and egg quality. However, the inclusion of 3% dried AMP (Sosnówka-Czajka and Skomorucha, 2021) in the diet tended to increase AEW, which may be related to its beneficial effects on body metabolism due to the higher proportion of AMP in the diet (corresponding to higher anthocyanins).

The nutritive value of eggs depends upon the composition of lipids, proteins, and other active substances. Lipid is the main component (30%) of egg yolk, which has a large content of fatty acids (Nielsen, 1998). C18:1n9-c and C16:0 are the most abundant fatty acids in egg yolks. In this trial, the addition of 0.5% FAMP reduced the percentage of C16:0 in egg yolks. The excess content of C16:0 may be harmful to the human body by increasing the production of harmful lipids, impairing cellular function, and inducing inflammation (Palomer et al., 2018). Meanwhile, FAMP supplementation decreased the SFA, especially 0.5% FAMP, which is partially in line with Selim et al. (2023), who found that supplementing grape pomace in laying hens' diets reduced the SFA. The CON group had a higher content of arachidonic acid in egg yolk, which may partly result from its increased biosynthesis of the unsaturated fatty acids pathway of cecal microbiota. Moreover, we found that the addition of FAMP (0.25%−0.1%) had beneficial impacts on the health lipid indices of egg yolk, which decreased the AI and TI while increasing the H/H, HPI, and DFA values. A relatively lower AI or TI value is beneficial to coronary artery health due to lower platelet aggregation (Woloszyn et al., 2020). The H/H index is defined as the Σhypercholesterolemic fatty acids/Σhypercholesterolemic fatty acids ratio and is also strongly associated with cholesterol metabolism. With respect to nutriology, the relatively higher H/H value is considered to have a greater potential to maintain physical health (Fernandes et al., 2014). The higher H/H and HPI (the reciprocal of AI) ratios provide protection against cardiovascular diseases (Hanus et al., 2018). The DFA index involves fatty acids, which are considered to have either neutral or cholesterol-lowering effects (Banskalieva et al., 2000). Thus, dietary FAMP supplementation positively affected the nutritive value of egg yolk. The optimal dosage was 0.5% FAMP, suggesting that FAMP had quadratic effects on fatty acid indices in egg yolk.

Egg albumen is rich in proteins, peptides, and amino acids (Sun et al., 2019), which possess antimicrobial, antioxidant, and anti-inflammatory functions (Lee and Paik, 2019). In the present study, the addition of 0.25% FAMP decreased the valine concentration, which may be related to its increased valine, leucine, and isoleucine degradation pathway of cecal microbiota. The addition of FAMP had no significant impact on most of the amino acid composition of egg albumen (Table 4). In contrast, a diet supplemented with 120–360 mg/kg anthocyanin-rich purple corn extract could improve most EAA and NEAA concentrations in eggs (Li J. et al., 2023). This disagreement is possibly influenced by the differences in feed composition, breeds, and ages of laying hens. For example, L-glutamine supplementation increased the levels of asparagine, phenylalanine, tryptophan, and tyrosine of albumen (Tomaszewska et al., 2021).

The growth and the role of ovarian follicles affect the laying performance of layers (Song et al., 2023), which is closely related to hormone regulation (Brady et al., 2020). The aged laying hens exhibit abnormal secretion of steroid hormones (Wu et al., 2023). In our study, the inclusion of 0.25%−1.0% FAMP increased the white follicles and the ovarian FSH, LH, and E_2_ levels. The small white follicles are involved in the estradiol secretion (Huang et al., 2021). FSH stimulates 3b-hydroxysteroid dehydrogenase (HSD3B1) production in granulosa cells by combining it with its receptor (FSHR) (Huang et al., 2021). Our findings suggested that the addition of 1% FAMP increased ovarian *HSD17B1* expression. HSD17B1 catalyzes the transformation process of estrone into estradiol (the most active type of estrogen), which is responsible for testosterone generation (Ruan et al., 2023). These findings indicate that FAMP supplementation could enhance ovary function by regulating follicle development and hormone synthesis.

Yolk precursors formed in the liver are transported into the ovary of laying hens through blood circulation (Li et al., 2015). Estrogen stimulates the synthesis of apo very low-density lipoprotein II (ApoVLDL II) and apolipoprotein B (APOB) and the induction of hepatic very low-density lipoprotein (VLDL) assembly procedures for VLDL Y (VLDLy) formation (Walzem et al., 1999). ApoVLDL-II prevents the lipolytic action of lipoprotein lipase on VLDLy for lipid deposition in the oocyte (Schneider et al., 1990). Aged laying hens tend to have abnormalities in their LBO axis function (Amevor et al., 2021). Our findings showed that genes related to yolk precursors synthesis (e.g., *ACC, FAS*, and *SCD*) and transport (e.g., *VTG II, ApoVLDL II*, and *APOB*) of the liver were upregulated in aged laying hens after FAMP supplementation, which could coordinately generate yolk lipids (Amevor et al., 2021). Li et al. observed that the *ESR2* expression in the liver was significantly upregulated in hens with higher laying performance (Li et al., 2015). We found that 1.0% FAMP increased *ESR2* expression in the liver. Similarly, the addition of 1.0% and 4.0% *Aronia melanocarpa* increased the levels of estrogen and proteins as manifested by yolk precursors in the liver of laying hens (Jing et al., 2022). Collectively, the addition of FAMP may contribute to the yolk lipid deposition in the ovary by regulating the function of the LBO axis.

Oxidative stress is a critical cause of ovarian recession, accompanied by decreased laying performance and egg quality of aged hens (Bao et al., 2022). In this study, 0.5% FAMP supplementation increased the ovarian T-AOC and SOD levels, which belong to the enzymatic defense system of the body (Wu et al., 2023). AMP contains total polyphenols, which have antioxidant potential (Sidor and Gramza-Michalowska, 2019). Dietary 2.5% AMP increased tocopherol content and exhibited better antioxidant properties in egg yolk (Loetscher et al., 2014). Polyphenol-rich additives like AMP, *Aronia melanocarpa* (Jing et al., 2022), grape pomace (Reis et al., 2019), and purple corn extract fed to laying hens (Li J. et al., 2023) improved ovary or plasma antioxidant capacity via regulating the Keap1/Nrf2 signaling pathway, which is responsible for xenobiotic and oxidant elimination (Bhattacharyya et al., 2014). Nrf2 regulates defensive gene expression concerning antioxidant proteins, such as HO-1, GPX1, SOD1, and SOD2, which exert antioxidant effects (Tonelli et al., 2018). Keap1, a negative regulator of Nrf2, contributes to its retention in the cytosol. The present study showed that supplementation of 0.5% FAMP in layer diets upregulated the *Nrf2* and downregulated the *Keap1* expressions in the ovary. Our results suggest the addition of FAMP could enhance the antioxidant function of aged laying hens.

Intestinal microbiota communities and their metabolites are essential for the host physiology metabolism. The cecum has been regarded as the main site for containing relatively higher dominant microbial communities in laying hens (Ricke et al., 2022). The present study revealed that the addition of 0.25% FAMP increased the α-diversity of cecal microbiota, including richness indexes (Observed_species and Chao1) and the evolutionary diversity index (Faith-pd), but decreased the coverage index (Goods coverage), indicating that laying hens fed with 0.25% FAMP has the higher species abundance in the cecum. FAMP supplementation resulted in a difference in the β-diversity index, suggesting the addition of FAMP could alter the cecal microbiota composition. In line with previous studies, Bacteroidetes and Firmicutes were the predominant phyla of aged laying hens (Dai et al., 2022). The decline in the value of Firmicutes/Bacteroidetes may be associated with intestinal inflammation (Stojanov et al., 2020). The addition of 1% FAMP had higher Firmicutes and lower Bacteroidetes abundance, suggesting the addition of 1% FAMP was beneficial for the gut health of the laying hens. Dietary 1% FAMP supplementation also increased probiotic species such as *Pediococcus, Lactobacillus*, and *Acetobacter*, which can produce lactic acid and SCFA and modulate the elements of the gut–liver axis (Yu et al., 2021; Wen et al., 2023). Meanwhile, *Lactobacillus* was more abundant in laying hens with high laying performance (Wang et al., 2020). In addition, Proteobacteria is considered a potential diagnostic criterion for dysbiosis and other diseases (Shin et al., 2015). In the present study, the addition of FAMP (0.25%−1.0%) significantly decreased the Proteobacteria abundance, including *Methylobacterium* (Kovaleva et al., 2014), *Ralstonia* (Ryan and Adley, 2013)*, Shigella* (Feng et al., 2021), and *Acinetobacter* (Maslova et al., 2022), which can be opportunistic pathogens. Additionally, there is an interplay between the cecal microbiota and phenotypic indicators (e.g., metabolites) of egg yolk (Tian et al., 2024). In this study, *Methylobacterium* and *Shigella* showed positive correlations with SFA, AI, and TI, and those also presented negative correlations with H/H, HPI, and DFA. Thus, FAMP could improve egg yolk health indexes, which may be related to reducing harmful bacteria.

The SCFA are the main metabolites of dietary fiber fermented by gut microbiota, which participate in energy and lipid metabolism and maintain gut homeostasis (He et al., 2020). Our findings show that the addition of 1% FAMP increased cecal acetate, propionate, butyrate, and valerate concentrations, which may be closely associated with the changes in microbial community structure. For instance, butyrate and Firmicutes (a butyrate-producing bacteria) were uniformly enhanced in the 1% FAMP group. Meanwhile, the pathway of butanoate metabolism and propanoate was also enriched in the 1% FAMP group by the PICRUSt2 analysis of microbiota. It is consistent with the fact that the 4% AMP supplementation increased the cecal acetate and butyrate concentrations in growing pigs (Ren et al., 2022). Therefore, the above findings suggest that the addition of FAMP contributes to increasing the cecal SCFA content, which may also have positive effects on gut health.

## 5 Conclusion

**Figure 7:**
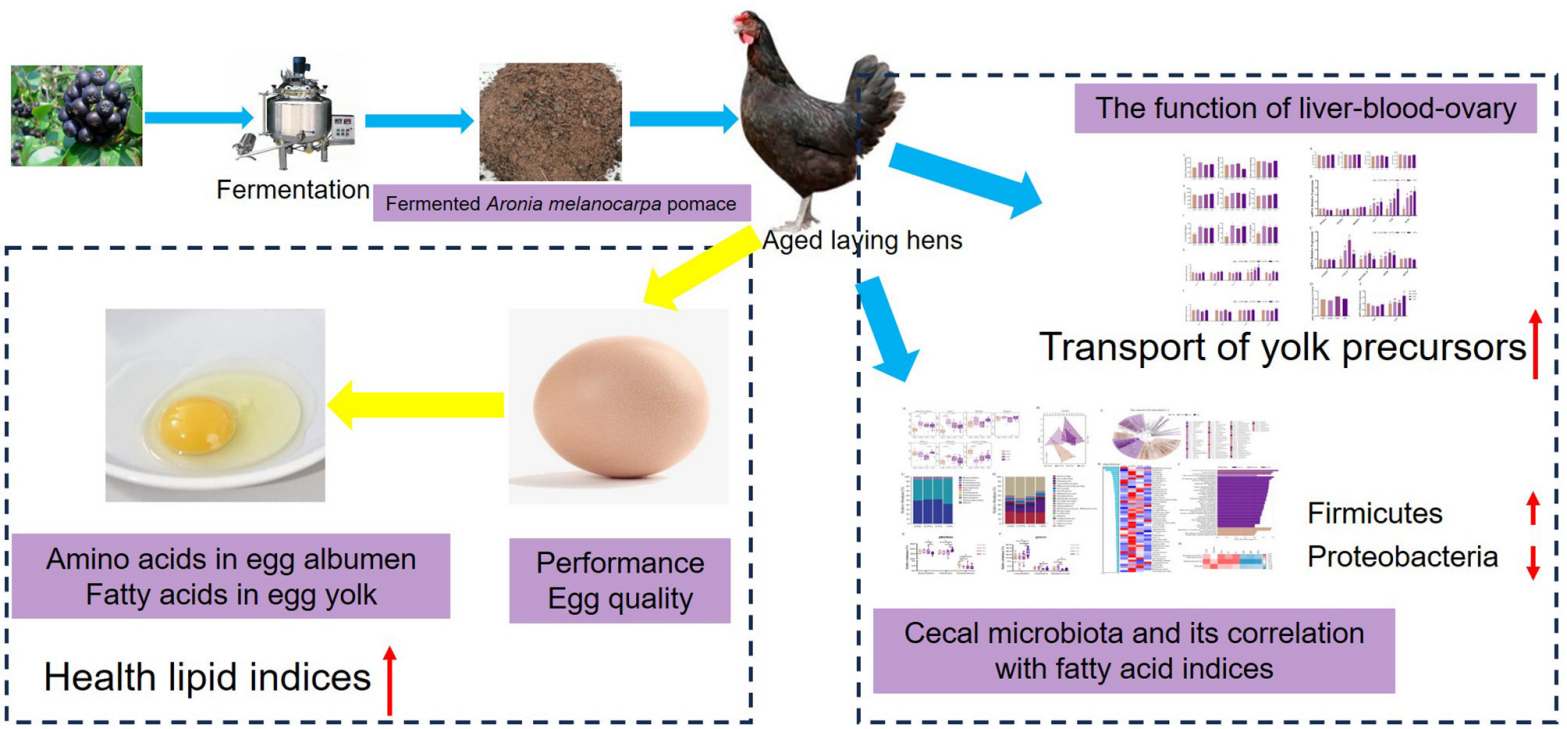
Effects and potential mechanisms of fermented *Aronia melanocarpa* pomace on performance, quality, and nutritive value of eggs, ovarian function, and cecal microbiota composition of aged laying hens.

The inclusion of FAMP in aged laying hen diet improved the health lipid indices in the egg yolk by decreasing the total SFA, AI, and TI indices, as well as increasing the H/H, HPI, and DFA indices. The addition of FAMP improved ovarian function by regulating the yolk precursor formation in the LBO axis and enhancing the antioxidant function of aged laying hens. Meanwhile, FAMP addition could regulate the cecal microbiota composition and increase SCFA concentrations of aged laying hens to promote gut health (Figure 7). Our findings provide fundamental evidence for the utilization of FAMP in aged laying hens due to its positive effects on the nutritive value of egg yolks by improving the LBO function and microbiota composition.

## Data availability statement

The original contributions presented in the study are publicly available. This data can be found here: Science Data Bank, https://doi.org/10.57760/sciencedb.09193.

## Ethics statement

The animal study was approved by the Animal Care and Use Committee of the Institute of Subtropical Agriculture, Chinese Academy of Sciences. The study was conducted in accordance with the local legislation and institutional requirements.

## Author contributions

ZL: Conceptualization, Data curation, Formal analysis, Investigation, Methodology, Visualization, Writing – original draft. BQ: Investigation, Writing – review & editing. TC: Investigation, Writing – review & editing. XK: Conceptualization, Funding acquisition, Project administration, Writing – review & editing, Writing – original draft. QZ: Validation, Writing – review & editing. MA: Validation, Writing – review & editing. YC: Supervision, Writing – review & editing. WL: Supervision, Writing – review & editing. QH: Conceptualization, Funding acquisition, Writing – review & editing.

## Supplementary material

Supplementary Table S1 Amino acid composition of the fermented *Aronia melanocarpa pomace*.

Supplementary Table S2 Fatty acid composition of the fermented *Aronia melanocarpa* pomace.

Supplementary Table S3 Composition and nutrient levels of basal diet.

Supplementary Table S4 Specific primers for PCR analysis.
