# Supplementary material for: Fermented Aronia melanocarpa pomace improves the nutritive value of eggs, enhances ovarian function, and reshapes microbiota abundance in aged laying hens
Source: Front Microbiol. 2024 Jun 19;15:1422172. doi: 10.3389/fmicb.2024.1422172 (PMC11220260; doi:10.3389/fmicb.2024.1422172)
Supplement: Supplementary Table S1 — Amino acid composition of the fermented Aronia melanocarpa pomace. [file Table_1.DOCX]

**Supplementary information for**

**Performance, egg quality, yolk fatty acid profile, and microbiota composition of laying hens fed fermented *Aronia melanocarpa* pomace**

**Zhihua Li^1,2^, Binghua Qin^2^, Ting Chen^2^, Xiangfeng Kong^2^*, Qian Zhu^2^, Md. Abul Kalam Azad^2^, Yadong Cui^3^, Wei Lan^3^, Qinghua He^1^***

^1^Department of Food Science and Engineering, College of Chemistry and Environmental Engineering, Shenzhen University, Shenzhen, 518071, China

^2^Hunan Provincial Key Laboratory of Animal Nutritional Physiology and Metabolic Process, National Engineering Laboratory for Pollution Control and Waste Utilization in Livestock and Poultry Production, Institute of Subtropical Agriculture, Chinese Academy of Sciences, Changsha, 410125, China

^3^School of Biology and Food Engineering, Fuyang Normal University, Fuyang, 236037, China

*** Corresponding:**

Dr. Qinghua He

[qinghua.he@szu.edu.cn](mailto:qinghua.he@szu.edu.cn)

Dr. Xiangfeng Kong

[nnkxf@isa.ac.cn](mailto:nnkxf@isa.ac.cn)

**Table S1.** Amino acid composition of the fermented *Aronia melanocarpa* pomace (%, as fed-basis)

| Items | Content | Items | Content | Items | Content |
| --- | --- | --- | --- | --- | --- |
| Alanine | 0.49 | Histidine | 0.20 | Isoleucine | 0.38 |
| Arginine | 0.52 | Isoleucine | 0.38 | Leucine | 0.65 |
| Aspartate | 0.93 | Leucine | 0.65 | Threonine | 0.38 |
| Cysteine | 0.17 | Lysine | 0.46 | Tyrosine | 0.22 |
| Glutamate | 1.68 | Methionine | 0.13 | Valine | 0.47 |
| Glycine | 0.55 | Phenylalanine | 0.44 | Total amino acids | 8.48 |

**Table S2.** Fatty acid composition of the fermented *Aronia melanocarpa* pomace (% of total fatty acids)

| Items | Content | Items | Content |
| --- | --- | --- | --- |
| Lauric acid C12:0 | 0.42 | Linoleic acid C18:2n-6c | 55.97 |
| Myristic acid C14:0 | 0.37 | α-Linolenic acid C18:3n-3 | 2.11 |
| Palmitic acid C16:0 | 11.61 | Arachidic acid C20:0 | 0.83 |
| Palmitoleic acid C16:1n-7c | 0.34 | 11c,14c-eicosadienoic acid C20:2n-6 | 2.75 |
| Stearic acid C18:0 | 2.53 | Docosanoic acid C22:0 | 0.50 |
| Oleic acid C18:1n-9c | 22.16 | Tetracosanoic acid C24:0 | 0.41 |

**Table S3.** Composition and nutrient levels of basal diet (%, as fed-basis)

| Ingredients | Content (%) | Nutrients | Level (%)^2)^ |
| --- | --- | --- | --- |
| Corn | 64.20 | Metabolizable energy (MJ/kg) | 11.38 |
| Soybean meal | 21.60 | Crude protein | 15.56 |
| Limestone | 8.00 | Ether extract | 5.10 |
| Soybean oil | 1.20 | Crude ash | 11.00 |
| Premix^1)^ | 5.00 | Calcium | 3.52 |
| Total | 100.00 | Total phosphorus | 0.42 |
|  |  | Methionine | 0.33 |
|  |  | Lysine | 0.95 |

^1^Premix supplied per kilogram of diet: Vitamin A, 10,000 IU; Vitamin D_3_, 3000 IU; Vitamin E, 20 IU; Vitamin K3, 1.75 mg; Vitamin B_1_, 2 mg; Vitamin B_2_, 6 mg; Vitamin B_6_, 3 mg; Pantothenic acid, 8.5 mg; vitamin B_12_, 0.02 mg; Nicotinamide, 40 mg; Folic acid, 1 mg; Biotin, 0.24 mg; Choline chloride, 450 mg; Methionine, 1.3 g; Lysine, 0.95 g; Calcium, 5 g; Phosphorus, 0.75 g; Copper, 8 mg; Fe, 75 mg; Mn, 100 mg; Zn, 65 mg; I, 0.8 mg; Se, 0.3 mg; sodium chloride, 3 g.

^2^ Metabolizable energy was calculated value, and other nutrient levels were measured values.

**Table S4.** Specific primers for PCR analysis

| Gene name | GenBank accession no. | Sequence (5′→3′)^2^ | Product size (bp) |
| --- | --- | --- | --- |
| *β-actin* | NM_205518.1 | F: ATGAAGCCCAGAGCAAAAGA  R: GGGGTGTTGAAGGTCTCAAA | 223 |
| *ACC* | NM_205505.1 | F: GTTGTGGTTGGCAGAGCAAG  R: GCACCAAACTTGAGCACCTG | 284 |
| *APOB* | NM_001044633.2 | F: AAATGTCCAAGGTGCAGCAG  R: CAATCAGCTTCCCGTTACCG | 185 |
| *APOVLDL II* | NM_205483.3 | F: TAGCACCACTGTCCCTGAAG  R: TTTTCCATCAGCTGTTCCGC | 237 |
| *CYP11A1* | NM_001001756.2 | F: CAGAGGCACAGCAGTTCATC  R: CACAAGGAGGCTGAAGAGGA | 239 |
| *CYP17A1* | NM_001001901.3 | F: TGCTTCAACTCCTCCTACCG  R: CATCAGGTCCCTCACAGTGT | 243 |
| *CYP19A1* | NM_001001761.4 | F: TGAGAGTTTGGATCAGCGGT  R: ACAAGACCAGGACCAGACAG | 220 |
| *ESR1* | NM_205183.2 | F: AATGAACAGGGCAGCTTTGG  R: CTCACAAGACCAGACCCCAT | 210 |
| *ESR2* | NM_204794.3 | F: CTCAGCACAGTCAGTCCAGA  R: AGACTTCCATCCAGCAGCTT | 238 |
| *FAS* | NM_205155.3 | F: TGAAGGACCTTATCGCATTGC  R: GCATGGGAAGCATTTTGTTGT | 96 |
| *FSHR* | NM_205079.2 | F: GTAGCATGTCTCCGGCAAAG  R: GAAGGCATCAGGTTTGGGTG | 185 |
| *GPX1* | NM_001277853.2 | F: ATGTTCGAGAAGTGCGAGGT  R: AGTTCCAGGAGACGTCGTTG | 160 |
| *HO-1* | NM_205344.1 | F: ATGCCTACACCCGCTATTTG  R: ATCTCAAGGGCATTCATTCG | 178 |
| *HSD3B1* | NM_205118.2 | F: GGCTGCTGGACAAAGACTTC  R: GCCCAAGGTGTCAATGATGG | 173 |
| *HSD17B1* | NM_204837.1 | F: GCAGTGTTTGAGGTGAACGT  R: CATGTGGATGTTGAAGGGCC | 216 |
| *Keap1* | MN416132.1 | F: CATCAACTGGGTGCAGTACG  R: AGGGTGAGGTCCTGGAAGAT | 183 |
| *LHCGR* | NM_204936.2 | F: ATCTCCCAGAGTGACTCCCT  R: TCTGCGTCAAGTCTGGGAAT | 193 |
| *MTTP* | NM_001109784.3 | F: CCCATTAGCGTCGTGAAAGG  R: CCGGTTCTTGACGTTGGTTT | 174 |
| *Nrf2* | NM_205117.1 | F: CCACCCTAAAGCTCCATTCA  R: ATTCTTGCCTCTCCTGCGTA | 217 |
| *PPAR-α* | NM_001001464.1 | F: CAAACCAACCATCCTGACGAT  R: GGAGGTCAGCCATTTTTTGGA | 64 |
| *PPAR-γ* | NM_001001460.2 | F: AGGGCGATCTTGACAGGAAA  R: TAATCTCCTGCACTGCCTCC | 190 |
| *SCD1* | NM_204890.1 | F: CACCACCACTGTCACCTCAC  R: CCCAATAATGGCCCCTAGAT | 214 |
| *SOD1* | NM_205064.1 | F: ATTACCGGCTTGTCTGATGG  R: CCTCCCTTTGCAGTCACATT | 173 |
| *SOD2* | NM_204211.1 | F: CCTTCGCAAACTTCAAGGAG  R: CCAGCAATGGAATGAGACCT | 162 |
| *SREBP1* | AY029224.1 | F: GTCGGCGATCCTGAGGAA  R: CTCTTCTGCACGGCCATCTT | 105 |
| *VLDLR* | NM_205229.2 | F:AGCTGGGTGTGTGATGATGA  R: GCAGTTCCCATCTTCACACC | 246 |
| *VTG II* | NM_001031276.2 | F: CTCGCTGCTCAGTTTCGTAC  R: CTCTCAGCCCCATCTACCAG | 247 |

*ACC*, acetyl-CoA carboxylase; *APOB*, apolipoprotein B; *ApoVLDL II*, apo very low density lipoprotein II; *CYP11A1*, cytochrome P450 family 11 subfamily A member 1; *CYP17A1*, cytochrome P450 family 17 subfamily A member 1; *CYP19A1*, cytochrome P450 family 19 subfamily A member 1; *ESR1*, estrogen receptor 1; *ESR2*, estrogen receptor 2; *FAS*, fatty acid synthase; *FSHR*, follicle stimulating hormone receptor; *GPX1*, glutathione peroxidase 1; *HO-1*, heme oxygenase 1; *HSD3B1*, 3 beta- and steroid delta-isomerase 1; *HSD17B1*, hydroxysteroid 17-beta dehydrogenase 1; *Keap1*, Kelch-like ECH-associated protein 1; *LHCGR*, luteinizing hormone/choriogonadotropin receptor; *MTTP*, microsomal triglyceride transfer protein; *Nrf2*, NF-E2-related factor 2; *PPAR-α*, peroxisome proliferator activated receptor-alpha; PPAR-γ, peroxisome proliferator activated receptor-gamma; *SCD1*, stearoyl-CoA desaturase 1; *SOD1*, superoxide dismutase 1; *SOD2*, superoxide dismutase 2; *SREBP1*, sterol regulatory element binding protein 1; *VLDLR*, very low density lipoprotein receptor; *VTG II* , vitellogenin 2.
